# Report of Observations Made in the British Military Hospitals in Belgium, after the Battle of Waterloo; with Some Remarks upon Amputation

**Published:** 1817-03

**Authors:** 


### PART II.

### COMPREHENSIVE ANALYTICAL REVIEW OF MEDICAL LITERATURE.

"Tros, tyriusve, nobis nullo discrimine agetur."

*Report of Observations made in the British Military Hospitals in Belgium, after the Battle of Waterloo*; with some Remarks upon Amputation. By JOHN THOMSON, M.D. F.R.S.E. &c. Octavo, pp. 281. London, 1816.

"*AFTER* the battle of Waterloo!" There is something chivalrous—almost romantic, in a metropolitan surgeon of England, Scotland, or Ireland, leaving his bed of down to traverse the field of [*after*] battle, and view those terrible sights which such a scene presents. Far be it from us to suspect that these philanthropic gentlemen did not cross the German Ocean for the entire purpose of pouring wine and oil into the wounds of our gallant countrymen. It would be illiberal in the extreme, to impute to them any motives of self-interest—any wish to monopolize information—any design to *make* a book—any ambition to enrich a lecture with the wounds of Waterloo. If those who stood the brunt of that bloody day, and toiled without rest or sleep for weeks afterwards, are unable to record the deeds which they performed, the phænomena which they witnessed—then Dr. Thomson has, and Mr. Bell will have, conferred a benefit on medical society.*

If the writers and teachers of the day had reaped the professional harvest which rotted in the vallies of Spain, or were buried in the waters of the Atlantic, or the Nile, what volumes of naval and military surgery might now have enriched our libraries! A false delicacy, a foolish pride, or a culpable negligence, has certainly deprived the medical world of inestimable treasures, which are now irre-

* We have heard that Mr. Bell was four days in the Vicinity of Waterloo.

trievably lost in the gulph of time. Without any fastidious investigation, therefore, of the motives which led to, or the justice which governed the publication of the volume before us, we shall faithfully delineate its contents; in the confident expectation that more ample details will yet be laid before the world, of this memorable battle; and that the Podalirii and Machaons of Waterloo will be recorded on the rolls of fame, as long as science lifts her head over ignorance, or medicine is cultivated as the noblest of arts.

In the tremendous conflicts of the 16th and 18th of June, nearly two thousand were killed and eight thousand wounded on the part of the British army. The weather was stormy and rainy during the 17th and 18th, but the men did not appear to receive any injury thereby, though many were unavoidably left on the field for some days. The most indefatigable exertions were inadequate to afford immediate attention to every case. The labours of the medical officers were continued without intermission for several days and nights, with scarcely an interval for rest or refreshment!

The inhabitants of Brussels and Antwerp exhibited noble traits of humanity and generosity in their attention to our wounded. The Belgians had exhibited the deepest sense of interest in the success of the British from the beginning of the battle, and the heroism which delivered them so suddenly by a victory so decisive, excited in their minds the strongest emotions of admiration and sympathy. The Brusselese, for some days after the action, devoted themselves entirely to the care of the sick, and eagerly received them into their houses. On the day after the battle, the streets and squares presented a most singular and interesting spectacle. The shops were shut, the people were at their doors administering cordials and dressings to the wounded. The most delicate females and people of rank were thus employed. Hundreds of wounded were seen in the streets, and every house contained some. The inhabitants made the greatest sacrifices with cheerfulness of their accommodations and personal services to their wounded guests, whom they liberally supplied with wine, fruit, and other luxuries. This kindness, of course, proved, in many instances, injurious; but it was an amiable failing.

It was impossible for sick or wounded men to be better lodged, or more amply supplied with every thing necessary for their situation. The wards of the hospital were large and well aired; the beds excellent; provisions good, cheap, and abundant; fruits in plenty; the inhabitants kind, and the medical officers unremitting in their duties. The wounded themselves maintained their character on the bed of sickness and death! They bore their sufferings with a fortitude and patience worthy of the heroes who had fought and conquered in the battle of Waterloo!

### Different Kinds of Wounds.

1. *Incised Wounds.* These were principally by the sabre, and situated on the head, temples, face, back of the neck, and shoulders; from the retraction of their edges, many of these presented frightful appearances. Adhesion by the first intention is universally attempted by the English, but not so by the French surgeons. The English, perhaps, err in leaving no interstices between the adhesive straps. The stitch is now entirely laid aside.

2. *Punctured Wounds.* These were principally by the *lance*, a few only by the *bayonet*. The *former* cuts more, occasions greater hæmorrhage, and is probably more deadly than the *latter*. The lance-wounds heal readily, and occasion little symptomatic fever. Yet, sometimes, even here, the inflammation was propagated along continuous membranes, with formation of matter beneath the fasciæ —two of the most remarkable local phænomena attendant on the progress of punctured wounds. Tetanus rarely supervened here; and when it did, it was generally of a chronic nature.

3. *Contused and lacerated Wounds.* These were occasioned by cannon balls, cannister shot, and pieces of bombs. The effects of these vary according to the velocity, size and form of the impinging body; and to the structure and functions of the part receiving the impulse. The stop which is sometimes put to the flow of blood through the larger arteries is a well known phænomenon of lacerated wounds. A man had his leg shot off by a cannon ball: in amputating the limb above the knee, the arteries bled not; neither did they require the ligature. A similar case occurred near the shoulder-joint. Mr. Guthrie dissected a limb, cut off in consequence of mortification of the foot and leg, produced by a contusion from a cannon ball on the posterior part of the leg, the popliteal artery was found closed in the ham by coagulable lymph, which seemed to have proceeded from a rupture of the internal coat of the artery. The same gentleman saw an instance in Spain where a bullet had passed between the femoral artery and vein, in the middle of the thigh. The artery was closed by a coagulum above and below the place. Gangrene affected the foot and leg. Dr. T. saw two cases where the pulsations at the wrist ceased, in consequence of balls passing thro' the lower and fore part of the arm, near the brachial artery. We ourselves lately saw an instance of loss of pulse at the wrist, which, we believe, never returned, in consequence of a fracture of the humerus, with a lacerated wound near the course of the brachial artery: the arm was preserved. Dr. T. thinks that the closure in these instances may have been produced either by the rupture of the internal coats of the arteries or the communication of inflammation to them.

4. *Gun-shot Wounds.* The majority seen were musket shot. Many examples of the singular directions which balls often take in their course through different parts of the body were observed. Dr. T. thinks, that from the *post mortem* appearances in some instances, there was reason to believe that balls which had entered the cavities of the head, thorax, and abdomen, had taken the concave direction of the internal surface of their parietes, and ran for a considerable way between these parietes and the viscera which they contain. Of a ball split by the ege of the patella, one half passed through at the moment of the injury, the other remained in the joint without its presence being suspected. The most frequent example of division of balls was where they struck against the spherical surface of the cranium. Sometimes one portion entered, while the other passed exterior to the bone. Not unfrequently the balls lodged between the tables, flattened or unchanged in shape. Nothing but a sight of the ball itself can prove that it has not split, when once it has touched a bone. All idea of poisoned balls is now ridiculed, and with it the dilating, cauterizing, and sucking of wounds for the purpose of extracting poison. The dilatation of gun-shot wounds, on other accounts, is still an unsettled point; and Dr. T. thinks that Mr. Hunter's directions are yet the best.

5. *Hæmorrhage* is the great source of immediate danger, and probably the principal cause of death on the field of battle. Modern surgery shines in this department. The largest arteries are now fearlessly laid bare and tied with small but strong ligatures. The long continued pressure of a tourniquet is now unknown, and even for temporary purposes, the finger or pad is generally used by bold surgeons. We do not agree with our author, however, in saying, that for temporary suppression, the tourniquet is only used "by ignorant or timid surgeons."

Of secondary hæmorrhage, the first variety is that where the recently closed mouths of arteries are forcibly opened by an increased determination of blood to that part—occurring usually from the first to the fifth day. 2d. From the sloughing of arteries, (a very frequent occurrence in gun-shot wounds) from the fifth to the tenth day. 3d. From ulceration of the coats of arteries, occurring at all and very remote periods. 4th. There is a species of secondary hæmorrhage which takes place from the extremities of stumps after amputation, and from the canals of gun-shot wounds, from the 20th to the 35th day. It is always preceded by heat, pain, and throbbing in the surface from which it proceeds, generally occurring in sanguine and plethoric habits; after indulgence in nourishing or stimulating food, and bearing a strong resemblance to those spontaneous hæmorrhages which take place from the capillary vessels that open every where upon mucous surfaces. It seems to be produced by the same causes, and prevented or alleviated by the same antiphlogistic treatment. Dissection was unable to discover, even with the aid of injections, the vessels from which the blood had been poured in profusion before death.

Several instances, and some fatal ones, of the *first* kind of hæmorrhage, had taken place after the battle of Waterloo. Dr. T. has notes of more than fifty cases of secondary hæmorrhage, principally after the 20th day. The causes were not accurately ascertained; but, in a majority of cases, it appeared to arise from sloughing and ulceration of arteries, occasioned by hospital gangrene, and from the increased determination of blood into the capillary vessels upon the surfaces of wounds:—the hæmorrhagic effort occurring particularly among those who had obtained too liberal an allowance of wine and animal food.

### Wounds of the Head.

The injuries of the integuments had chiefly been inflicted by the sabre and musket balls, and healed rapidly; erysipelas rarely supervening. In some instances, portions of the cranium, and even the dura mater had been removed without much injury. In one case of the latter kind, a tendency to protrusion of the brain took place during an attack of inflammation, but subsided with the inflammation. This protrusion was not observed in any other instance. In a remarkable sabre cut, where an inch in breadth of the inferior part of the left lobe of the cerebellum was exposed, and seen pulsating for a period of eight weeks, no protrusion took place. Many were the cases where the external table only of the skull was divided by the sabre; and no inflammation communicated to the brain or membranes. In other cases, both tables were divided, and the edges of the internal turned in on the brain. In most of these, exfoliations took place; often accompanied by paralysis of the upper or lower extremities. Where musket-balls had struck the cranium, contusion of the substance of the brain was generally produced, even where no fracture or depression of the bone occurred. In a man who died in consequence of a blow from a musket-ball on the left side of the cranium, the contusion communicated through the whole breadth of the hemisphere, above the lateral ventricle, and the substance of the brain appeared in this track as though a bullet had passed through it.

Stupor in many instances continued after the compression of bone, or extravasated fluids, had been taken off. Some remarkable changes in the state of the pulse were observed in injuries of the head. In a wound of the posterior part of the skull with depression, the pulse had at one time sunk to 36. He seemed to recover from this state, and to do well; but extensive protrusion of the brain taking place, he died at the end of six weeks. The whole hemisphere of the brain was found converted into a soft red substance. In a man who had received a concussion, the pupil of the right eye was dilated, but not the left. Strabismus was a common attendant on injuries of the head.

Two kinds of secondary inflammation succeed gun-shot wounds; the confined, and the diffused. The former often admits of relief by operation; not so the secondary inflammation, which is diffused over the brain and its membranes. Unfortunately, dissection shews this last more frequently than the former: few examples, however, occurred in Belgium, owing probably to the very great pains which were taken by the military surgeons to enforce the antiphlogistic treatment and regimen in its fullest rigour. In a number of cases, where the cranium had been severely contused or fractured by musket-balls, fungous growths took place through the openings which had been made, both by the ball and the trepan. In a fracture of the vertex of the cranium, produced by a musket-ball, and followed by a fungus of the brain, paralysis took place in the lower extremities. In a case of wound made by a musket-ball on the right side of the forehead, and in which spiculæ of bone had been driven in upon the brain, a large fungus protruded. The formation of this fungus was followed by slow pulse, stupor, dilated pupils, slight strabismus, and distortion of the mouth. In the progress of this case, portions of the fungus had frequently been removed by escharotics and the hands of the patient, with apparent alleviation of the symptoms. Dissection shewed nearly the whole of the right hemisphere of the brain converted into a soft pulpy mass. The left hemisphere presented only a vascular turgescence on its surface.

Though stupor and paralysis often attended depressions of bone and extravasation of fluids, yet various instances occurred where both these last were unaccompanied by the former symptoms. In one case, where the middle of the right parietal bone was fractured and considerably depressed by a ball, which was extracted on the 20th day, neither stupor nor paralysis appeared. In another, a musket-ball fractured and flattened the right parietal bone, and lodged between the tables of the skull. The inner table was much depressed, and yet no bad symptoms supervened. Convulsions succeeding injuries of the head, with portions of bone driven in upon the brain, are known to be most dangerous symptoms; yet in more than one instance recovery took place after the depressed portions of bone had been removed, during repeated bleedings and the use of the antiphlogistic regimen.

Three cases were seen in which balls had entered the cavity of the cranium, and passed, on its inner surface, along the upper and lateral parts of the brain; and these cases all terminated fatally. In one of these cases, the ball entered at the lateral part of the os frontis, and passed out at the edge of the occipital bone of the same side. The patient appeared to recover till the 21st day, when he became affected with rigors and suppurative fever, of which he died. In one singular case, in which a ball, entering behind the right temple, and passing backwards and downwards, had fractured the bones in its passage, the ball appeared to be lodged in the surface of the brain, over the tentorium cerebelli, from which place it was extracted on the 17th day after the infliction of the injury.

No bad symptom had manifested itself previously to the operation; and this man recovered under the strictest antiphlogistic regimen, with little or no constitutional derangement, except a slight tendency to inflammatory fever, which, upon one occasion, was induced by an incautious allowance of a small quantity of wine and animal food. No instance was recorded in which a patient survived a shot which had passed through the long or short diameter of the brain.

WOUNDS OF THE FACE AND NECK.

1. *Face.* Wounds in this region, by musket-balls, are seldom dangerous, but often distressing from the pain and subsequent deformity. *These kinds* of wounds were, for obvious reasons, most numerous among our Waterloo heroes. A frequent and lamentable species of injury was that which had occasioned blindness, by the passage of balls through or near to the eyes. In some, the vision was destroyed without any apparent injury of the eyeball itself. In one case, where the ball had passed through behind the eyes, from temple to temple, one eye was destroyed by inflammation, and the other affected by amaurosis. In a third, under precisely similar circumstances, both eyes were affected by amaurosis, but without iuflammation being produced. In a fourth, where the bullet had entered the face on the upper and *left* side of the nose, and passed out anterior to the right ear, there was amaurosis of the *right* eye. The *left* eye was similarly affected in a case where the ball had entered the *right* side of the nose, and come out before the left ear. Dr. T. saw eight or ten patients in whom musket-balls had passed through behind the eyes, from temple to temple, in all of which were great swelling, pain, and tension of the head and face. On a superficial view, the balls would have been thought to have entered the cranium; but Dr. T. thinks that this was not the case. From the course of some balls, however, the optic nerves were probably divided.

The number of cases was considerable, also, in which bullets had passed directly through the substance of one or both eye-balls. Some very frightful wounds had been made by canister or grape shot, that had carried away the nose and fore-part of the face, exposing the maxillary sinuses and cavities of the nostrils. In these wounds, as well as in those where the upper and lower jaw-bones were broken, numerous fractures were produced, which gave rise to tedious and painful exfoliations. Fractures of the lower jaw, upon one or both sides, were very common occurrences, and more or less distortion of the face was generally the consequence.

2. *Neck.* Here were many remarkable escapes. In numerous instances, the bullets must have passed quite close to the great vessels of the neck; but in none had the carotid artery or internal jugular been opened. In all probability, where these vessels were divided, the event was fatal on the field. In a considerable proportion of cases, the balls entered by the side of the face, passed within or through the body of the lower jaw, and came out on the side of the neck, at almost every point from the base of the lower jaw to the middle and upper part of the back. Some of the primary branches of the external carotid must often have been divided or injured. The *primary* hæmorrhage, in these cases, had not been alarming; and *secondary* only occurred in three instances. In one of these last, where the ball had passed through the mouth, the hæmorrhage was suppressed by compresses applied to the external and internal orifices. In the other two instances, it was deemed necessary to lay bare and tie the carotid artery. In one of these, the ball had passed through the pharynx and wounded the trachea: the hæmorrhage was completely restrained by the ligature on the carotid, but the man died four days after the operation, from matter gradually passing through the wound of the trachea into the bronchia, and ultimately producing suffocation. The other recovered. No unequivocal case of wounded œsophagus presented; but balls, in several instances, appeared to have passed through the pharynx. In a very singular case, the ball entered the left ear, passed across the fore-part of the neck, and was lodged under the skin at the lower part of the cervical region. In two cases, the lower jaw was shot away by cannon balls: in one, the patient died of secondary hæmorrhage; in the other, of extension of mortification.

In the case of a Frenchman, the soft parts under the base of the lower jaw had been carried away by a piece of grape shot, and a frightful opening made through the upper and fore part of the neck into the mouth. The discharge of saliva was copious and distressing. He was fed by a funnel; had a voracious appetite; but by the end of the eighth week he had become lean and emaciated from the loss of saliva.

Several wounds of the larynx had been made by musketballs; the voice was much impaired and hoarse; exfoliations of cartilage were taking place, and a state resembling phthisis commencing.

In various instances of face and neck wounds, paralysis of the shoulder, arm, fore-arm, and fingers, was induced by division of some of the cervical nerves. The paralysis was, in some cases, immediate, in others more remote from the receipt of the injury. It was generally accompanied by pain. In a singular case of partial paralysis of the arm, a musket-ball had entered over the left side of the trachea, an inch and half from the inner extremity of the clavicle; passed from before backwards, and seemed to be lodged under the scapula. The paralysis of the arm was followed, in about twenty-four hours, by loss of voice, frequent attacks of vomiting, and violent and painful spasms of the diaphragm, indicating probably the communication of inflammation from the canal of the wound to the more important nerves of the neck. He recovered.

### WOUNDS OF THE CHEST.

Recoveries here were very numerous. The greater number of wounds were from musket-balls. Except when air comes out during coughing, it is difficult to say whether wounds penetrate the sacs of the pleura. Hæmoptoe indicates lesion of the lung itself. A man in kneeling received a musket-ball, which entered at the upper and middle part of the sternum, ran along the surface of the bone, continued its course downwards, passed along in the parietes of the abdomen into the right side of the scrotum, from whence it was cut out. The long track of wound here healed readily, without the formation of abscess, or the extension of inflammation. Abscesses, however, do sometimes occur in such canals, even where neither wadding nor portions of clothes are driven in with the ball. A few instances occurred where, from such tracks of balls, though not penetrating the chest, inflammation spread to the thoracic viscera. In a remarkable case, the ball entered above the middle of the clavicle, and passed out at a point directly behind. Neither at first, nor subsequently, was there reason to believe that the ball had wounded the pleura. Inflammation, however, of this membrane came on, and terminated in suppuration. The operation for empyema was performed, and four pints of pus evacuated. Sequel not related. No instance is recorded where the internal mammary or intercostal arteries were wounded by pike or ball. In a gun-shot wound, however, secondary hæmorrhage, from an intercostal artery, took place on the 15th day, and was suppressed by a compress introduced into the wound, so as to press upon the open mouth of the vessel.

Hæmorrhage by the mouth had been more severe in wounds inflicted by the lance than by balls. In some few this hæmorrhage ceased entirely in twenty-four hours; but in a majority of cases it continued seven or eight days. When it passed this period, it ceased about the 14th day. Only one instance of longer duration occurred. The hæmorrhage from external wounds had often been very profuse; but always easily checked by pressure and closed orifices. In no case but one was this followed by inconvenience. This was a lance wound of the right side of the chest, from which a copious hæmorrhage took place, that was suppressed by compress and bandage. A sense of weight was next felt over this side, with difficulty of lying on the left side. The symptoms, however, were not so urgent as to require an operation.

In almost all the injuries of the chest attended with hæmorrhage, the lancet was freely employed by the English surgeons; which not only checked the flow of blood, but obviated the ulterior consequences — inflammation, &c. In a case where a musket-ball had entered the left shoulder, passed through the lungs, and come out below the left nipple, a profuse hæmorrhage of arterial blood from the mouth threatened immediate suffocation. The hæmorrhage was suppressed by repeated bleedings, pushed each time till relief was obtained. Leeches were applied to the side in great numbers, and the antiphlogistic plan strictly enjoined. Two hundred and fifty ounces of blood were abstracted in eighteen days, and recovery followed.

In three cases, balls had entered and lodged in the cavity of the thorax. The symptoms were severe, but convalescence was in progress. In one case, a considerable quantity of air and pus came through the wound on coughing. In a second, great difficulty of breathing had at one time occurred; but this yielded to blood-letting. In another case, the ball had entered below the angle of the left scapula; and the wound, through which blood, air, and pus had been discharged, healed. A dull sound, on percussion of the left side of the thorax, seemed to indicate effusion there, as there was also considerable difficulty of breathing. The patient, contrary to usual observation, lay best on his right side. In two cases, balls passed through the thorax from before backwards, lodged under the scapula, and elevated that bone. They saw ten cases where balls had entered the fore-part of the neck or chest, passed through the latter cavity, and made their exit through the scapula.

Three cases occurred where balls entered the chest through the left scapula. One came out near the left papilla; one close to the upper part of the sternum; one anterior to the outer edge of the right scapula. This patient spat blood for fifteen days, but had no great difficulty of breathing.

Two cases occurred where the ball had passed through both sides of the chest. In one, the ball entered on the lower part of the left side of the sternum, passed across under that bone, and came out below the right axilla. In the other, the ball entered below the left papilla, and passed out below and on the outer side of the right papilla. Pus flowed out of the left side of the chest upon coughing.

Very few cases of emphysema presented. Over a large lance-wound at the lower part of the left scapula, a circumscribed puffy tumour formed, attended with apparent enlargement of that side of the chest, and extreme dyspnœa. A free incision was made into this tumour, and air, which had been accumulated in great quantity in the sac of the pleura, escaped, together with a considerable quantity of bloody serum. Great and instantaneous relief ensued. Suppurations of the pleura are apt to follow wounds of the chest, if the inflammation be not promptly subdued, or if it return after being subdued. Hectic fever is too often the consequence. On opening two bodies that had died of emphysema, the lung, in one of these, was completely collapsed and agglutinated to the mediastinum, on the wounded side. The surface of the lung, as well as of the pleura costalis, was covered with a whitish purulent lymph. In the other, appearances in every respect similar had taken place, except that the wounded and collapsed lung was drawn across the chest, and attached by adhesion to one of the inner edges of the orifice of the wound on the parietes.

In several instances, the external wounds healing while pus was secreting internally, empyema followed. The operation was performed, with great and sensible relief, both at Brussels and Antwerp. In one case only, a portion of lung protruded through a wound in the chest. Here, though the communication of the external air with the cavity of the chest had been cut off by the adhesion of the protruding portion of the inner edge of the orifice, in the parietes, yet the patient suffered much from repeated attacks of inflammation.

Having finished the wounds of the thorax, we shall close our analysis for the present; reserving for our next number, the wounds of the other remaining regions of the body.

---

